# Evaluation of augmented reality guidance for glenoid pin placement in total shoulder arthroplasty

**DOI:** 10.1007/s11548-025-03444-8

**Published:** 2025-06-11

**Authors:** Taylor Frantz, Frederick van Gestel, Pieter Slagmolen, Johnny Duerinck, Thierry Scheerlinck, Jef Vandemeulebroucke

**Affiliations:** 1 Department of Electronics and Informatics (ETRO), Vrije Universiteit Brussel (VUB), Pleinlaan 2, 1050 Brussels, Belgium; 2 imec, Kapeldreef 75, 3001 Leuven, Belgium; 3 Department of Neurosurgery, Universitair Ziekenhuis Brussel (UZ Brussel), Laarbeeklaan 101, 1090 Brussels, Belgium; 4 Department of Orthopedic Surgery and Traumatology, Universitair Ziekenhuis Brussel (UZ Brussel), Laarbeeklaan 101, 1090 Brussels, Belgium; 5 Department of Radiology, Universitair Ziekenhuis Brussel (UZ Brussel), Laarbeeklaan 101, 1090 Brussels, Belgium; 6 Materialise NV, Technologielaan 15, 3001 Leuven, Belgium

**Keywords:** Shoulder arthroplasty, Computer-aided navigation, Augmented reality, Glenoid pin placement

## Abstract

**Purpose:**

Computer-aided navigation and patient-specific 3D printed guides have demonstrated superior outcomes in total shoulder arthroplasty (TSA). Nevertheless, few TSAs are inserted using these technologies. Head-worn augmented reality (AR) devices can provide intuitive 3D computer navigation to the surgeon. This study investigates AR navigation in conjunction with adaptive spatial drift correction toward TSA.

**Methods:**

A phantom study was performed to assess the performance of AR navigated pin placement in TSA. Two medical experts performed a total of 12 pin placements into phantom scapula; six were placed using an end-to-end AR-navigated technique, and six using a common freehand technique. Inside-out infrared (IR) tracking was designed and integrated into the AR headset to correct for device drift and provide tool tracking. Additionally, the impact of IR tool tracking, registration, and superposed/juxtaposed visualization techniques was investigated.

**Results:**

The AR-navigated pin placement resulted in a mean entry point error of 1.06 mm ± 0.64 mm and directional error of ${1.66^\circ \pm 0.65^\circ }$. Compared with the freehand technique, AR navigation resulted in improved directional outcomes ($p=0.03$), while entry point accuracy was not significantly different ($p=0.44$). IR tool tracking error was 1.47 mm ± 0.69 mm and ${0.92^\circ \pm 0.50^\circ }$, and registration error was 4.32 mm ± 1.75 mm and ${2.56^\circ \pm 0.82^\circ }$. No statistical difference between AR visualization techniques was found in entry point ($p=0.22$) or directional ($p=0.31$) errors.

**Conclusion:**

AR navigation allowed for comparable pin placement outcomes with those reported in the literature for patient-specific 3D printed guides; moreover, it complements the patient-specific planning without the need for the guides themselves.

## Introduction

The shoulder is the most dynamic joint in the human body, which contributes to an elevated risk of mechanical and degenerative disorders over time. The prevalence of surgical interventions of the shoulder due to fractures and degenerative disorders is increasing, specifically TSA. Between 2011 and 2017, the number of interventions has risen by over 100% to 63,845 in the USA and is predicted to increase to over 350,000 by 2025 [1–3].

Proper placement of prosthetic components in TSA is critical to long-term interventional success. Failure in achieving this can result in joint instability, impingement and/or component loosening, necessitating revision, where incidence is reported between 0.5% to 9.0% in reverse total shoulder arthroplasty [4].

During TSA, a central pin is placed through the glenoid in the longitudinal axis of the neck to guide the location and direction of the glenoid component. As such, its position is critical. However, accurate placement is not trivial, and most prosthetic systems rely on surgical templates and guides, combined with operative experience, to achieve this. Moreover, the use of longer screws in fixation, or patients with smaller scapula only narrow these margins [5, 6].

The role of surgical planning based on CT imaging (irrespective of the use of guides) is becoming mainstream due to the mentioned challenges. Execution of this preoperative planning is often carried out using patient-specific 3D printed guides or computer aided navigation (CAN) systems. Both improve accuracy and precision in the execution of such planning and have led to improved implant purchase and a decline in revisions [7–10].

Augmented reality (AR) allows for the superposition of computer-generated graphics onto the real world and is becoming more prevalent in CAN. The use of augmented reality head-mounted displays (AR-HMDs) allows surgeons to view preoperative medical imaging and planning data overlaid with the operating field. Compared to traditionally navigated procedures, this provides a more intuitive visualization of data and can reduce operative time [11–14].

Cost and technical limitations in tracking quality have often prevented the greater interventional adoption of AR-HMDs in CAN [13, 15–18]. The proliferation of off-the-shelf AR-HMD hardware has greatly undercut costs of de-novo development; however, technical limitations remain. Gregory et al. [19] demonstrated an early proof of concept AR-assisted TSA solution using a commercial AR-HMD to visualize manually registered CT and planning data. In their study, registration was maintained in space using the device’s simultaneous localization and mapping (SLAM) capabilities. Although helpful during surgery, out-of-the-box SLAM performance of such devices makes them unsuitable for surgical navigation due to spatial drift of visualized data and patient motion.

One approach taken toward improving AR stability is the inclusion of external tracking hardware into the AR-HMD workflow: a so-called outside-in approach toward tracking [20–23]. Conversely, this can be done using the onboard sensors of the AR-HMD itself, the so-called inside-out approach, thus negating the need for ancillary tracking hardware. This latter approach has been demonstrated in both orthopedic and neurosurgical use cases [24–27]. In both cases, such secondary tracking also provides a mechanism for precise 3D data collection, useful in patient-data registration and tool-localization tasks.

In TSA, Kriechling et al. [28, 29] demonstrated the efficacy of adapting commercial AR hardware with inside-out tracking of QR codes, achieving a mean glenoid pin placement deviation of 2.3 mm ± 1.1 mm and $2.7^\circ \pm 1.3^\circ$ in phantom experiments, and 3.5 mm ± 1.7 mm and $3.8^\circ \pm 1.7^\circ$ in cadaver experiments. Likewise, Gu et al. [30] achieved a pin placement error of 1.5 mm and ${2.4}^\circ$, and 2.5 mm and ${1.5}^\circ$ in phantom and cadaver studies, respectively. However, due to the complexity of their markerless photogrammetric drift correction, secondary compute infrastructure was required for real-time performance.

**Fig. 1:**
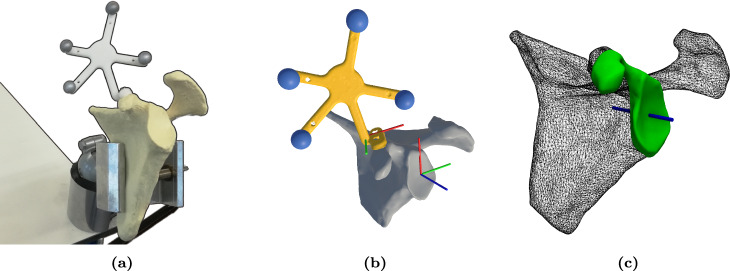
**a** Physical phantom tracker assembly clamped to desk. **b** Surface scan of the left scapula phantom with affixed tracker (yellow) and anatomical coordinate system (red–green–blue (RGB) axes) shown. **c** Planned trajectory into the phantom glenoid (blue), permissible sampling surface for registration (green)

Alternatively, as demonstrated in other surgical domains, data from depth and infrared reflectivity sensors may be used for the tracking of labeled trackers, providing a mechanism for sub-millimeter pose estimation of surgical hardware [31, 32]. Though this achieved excellent tracking performance, the method relies on multiple sensor streams (infrared and depth), potentially limiting deployability among off-the-shelf AR-HMDs. Moreover, the use of time-of-flight (ToF) depth sensor data for such tracking or registration as demonstrated by Gu et al. [22] and Li et al. [33] has demonstrated centimeter scale surface bias due to material infrared characteristics, requiring use case and material-specific calibration in order to be effective.

It is hypothesized that a computationally lightweight tracking solution integrated into a commercial AR-HMD can achieve similar or better performance relative to both standard practice and published literature in AR CAN TSA. Toward this, we present an integrated solution for adapting off-the-shelf AR hardware to guide central glenoid pin placement in TSA. The solution relies on an inside-out registration and tracking approach using standard surgical infrared retroreflective markers and the device’s integrated IR sensor only. We report the results of the performance during a phantom trial of registration, tracking, and end-to-end AR guidance.

## Materials and methods

### Experiment design

A non-arthritic scapula phantom no. 1021 (Sawbones, Vashon, WA, USA) was digitized using an LLP HD 3D scanner (Faro, Lake Mary, FL, USA), such that a closed 3D surface mesh was obtained. From this, a rigid tracker was designed to fit along the coracoid process, as this is typically accessible, and support a constellation of four retroreflective infrared markers (Northern Digital Inc., Waterloo, Ontario, Canada). The tracker was fabricated in PA-12 using selective laser sintering (EOS, Krailling, Germany) and affixed, with IR markers, to the phantom (Fig. 1a). This assembly was likewise digitized, resulting in a second surface mesh from which the 3D centroids of the IR markers were determined through sphere fitting (Fig. 1b).

Based on this surface mesh, a ground-truth glenoid trajectory was defined using Mimics (Materialise, Leuven, Belgium) and comprised an entry point and direction at the glenoid face (Fig. 1c). A coordinate system was defined representing anatomical directions at the entry point: superior-inferior (SI), anterior-posterior (AP), medial-lateral (ML).

A rigid tracker consisting of five passive IR markers was affixed to a System 7 surgical drill (Stryker, Kalamazoo, MI, USA) equipped with a 3.2 mm twist drill. Similarly to the phantom, the drill was 3D scanned, whereafter the coordinates of the IR markers were determined through sphere fitting. Additionally, and from the same scan, the position and orientation of the drill bit were identified. For all trackers, the coordinates of their respective IR markers were used to define a local coordinate system in which relevant data were referenced, e.g., a vector or point.

### AR application

To provide an AR workflow encompassing visualization, phantom registration, and drill guidance as described below, an AR application was developed in Unity3D (Unity Technologies, San Francisco, CA, USA), version 2019.4.40f1. The app was deployed to a HoloLens 2 (Microsoft, Redmond, WA, USA) AR-HMD and integrated an inside-out tracking algorithm for pose estimation of rigid trackers using the device’s short-throw IR sensor.

### Phantom registration

**Fig. 2:**
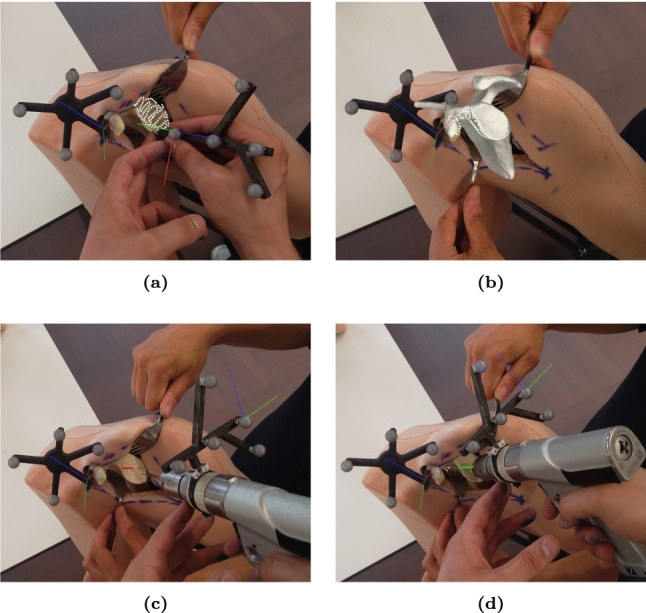
First person view of augmented reality (AR)-guided pin placement workflow during **a** point collection for registration, **b** visual assessment of final registration result, **c** alignment of the drill to the target trajectory, **d** drilling with the drill aligned to the planned trajectory

Registration between the virtual and physical phantom was achieved in two steps. First, the investigator used a handheld tracked stylus to identify four predefined points along the glenoid margin. These point pairs provided an initial alignment through least-squares registration. Next, the investigator collected additional points through tracing along the surface of the scapula, limited to regions accessible during deltopectoral approach (Fig. 1c). Final registration was achieved through an iterative closest point (ICP) method, minimizing the point-to-plane distance between the collected data and the scapular surface. Adding additional points allowed for refinement of registration until the investigator was visually satisfied with the alignment between the virtual and physical scapula (Fig. 2a, b).

**Fig. 3:**
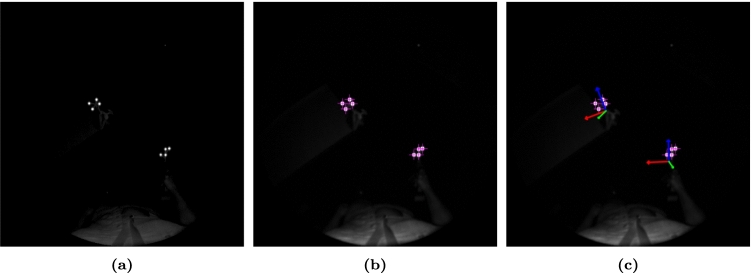
Image processing overview for infrared (IR) tool tracking. **a** IR reflectivity map from the HoloLens 2 device. **b** Detected IR markers (pink crosses). **c** Estimated 3D pose (red–green–blue axes) for both drill and coracoid tracker

**Fig. 4:**
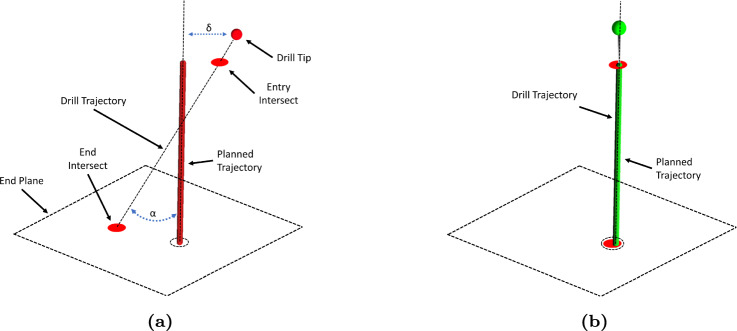
Visualization technique for augmented reality (AR) guided drilling, during **a** alignment of the drill to the target trajectory and **b** drilling of aligned drill to trajectory. Dashed lines are purely illustrative and not shown in the AR app

### Infrared tracking

Pose estimation of each IR labeled tracker was accomplished in four steps (Fig. 3). First, the IR reflectivity map of the HMD was acquired, Gaussian smoothed and histogram equalized based on the upper 20th percentile. Second, blob detection was used to determine the image position of illuminated IR markers. Then, using both the IR markers’ local coordinates within each physical tracker and their image positions, the pose in six degrees of freedom (DoF) was estimated using perspective-n-point geometry [34]. Lastly, this was transformed into the world coordinate system using the extrinsic transforms of the IR sensor relative to the HMD and world. Four IR markers are required for tracker detection, while tracking can be maintained with a minimum of three. A greater number of IR markers per tracker allow for partial occlusion of each tracker during the workflow.

### Scapular drilling

Drilling of the scapula was performed in two steps. First, the app guided the user to place the drill tip at a position upon the glenoid surface such that its distance to the trajectory ($\delta$) was minimized. Secondly, the drill was oriented about its tip such that it minimized its angle with respect to the planned trajectory ($\alpha$). To aid in alignment, two red dots were visualized on planes perpendicular to the planned trajectory’s start and end point, indicating intersection of the tracked drilling trajectory through both planes. When the drill achieved the correct position and orientation, the red dots, planned trajectory, and drill trajectory showed co-alignment (Fig. 4). During the positioning and orientation process, the color of the planned trajectory and stylus tool-tip changed independently from red to yellow and to green according to Eq. 1. The red/yellow colors indicate a “no-go” and the green color a “go” condition. This is appreciable in Fig. 2c, d during AR navigation.

1 $$\begin{aligned} color= {\left\{ \begin{array}{ll} \text {green}, &  \text {if}\ \alpha \le {2}^\circ \text { or } \delta \le {2}\,{\hbox {mm}} \\ \text {yellow}, &  \text {if}\ \alpha \le {4}^\circ \text { or } \delta \le {4}\,{\hbox {mm}} \\ \text {red}, &  \text {otherwise} \end{array}\right. } \end{aligned}$$

### Protocol

All measurements were performed by a surgical trainee and an expert in medical image processing, both having extensive experience with the AR-HMD hardware. For each evaluation, a new undrilled phantom scapula with a phantom skin envelope no.1509-24-2 (Sawbones, Vashon, WA, USA) was clamped into a support. The skin envelope was incised to provide an appropriate surgical field. Two drilling techniques were used: first, a control, relying on a proprioceptive guided freehand drilling technique based on prior imaging data and anatomical knowledge; and second, an end-to-end AR-navigated technique providing superposed drilling guidance, as described above, of registered planning. After drilling, a pin of 3.1 mm was inserted into the glenoid. Each of the two investigators performed each placement technique three times, resulting in 12 measurements.

In addition, a series of experiments were performed to investigate the contribution of system errors from tool tracking, phantom registration, and different navigation feedback positions upon the quality of the final pin placement.

**Fig. 5:**
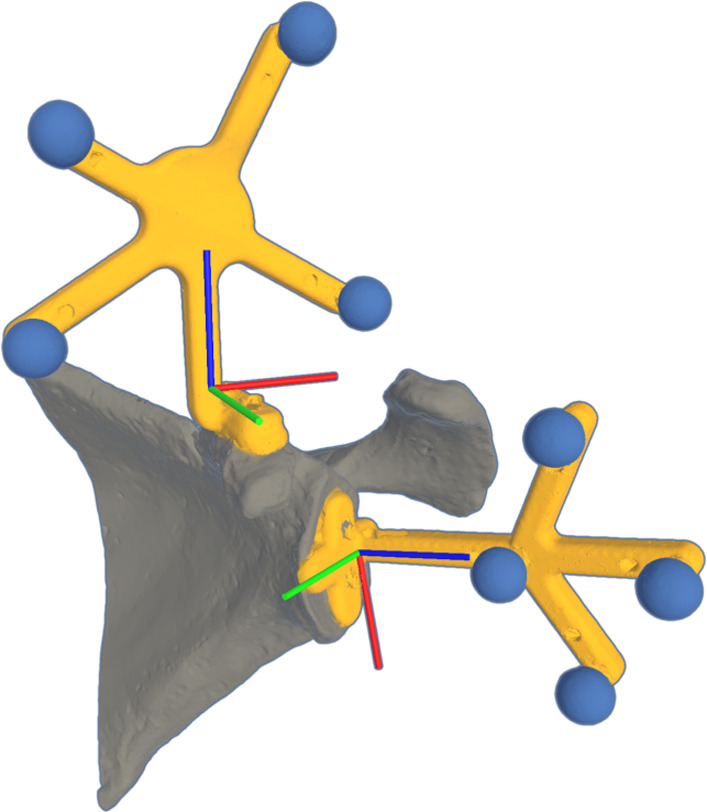
Phantom scapula for infrared (IR) tracking evaluation; trackers (yellow) with local coordinate systems (red–green–blue axes), passive IR retroreflective markers (blue)

To quantify IR tracking performance, a phantom scapula with an additional printed tracker was created (Fig. 5). This second tracker defined a specific anatomical coordinate system at the glenoid surface. During the experiment, the investigator was free to move about the proximity of the phantom while wearing the AR-HMD. Measured drift between the fixed spatial relationship of the trackers gives an estimate of the tracking uncertainty. As such, the pose for each tracker with respect to the AR world coordinate system was logged to the device for analysis a posteriori.

Registration quality was evaluated by comparing a series of registration outcomes between a moving AR scapula surface mesh and a fixed phantom scapula, as described above. The two investigators each performed eight registrations. For each attempt, the registered pose (6-DoF), with respect to the phantom tracker, was logged to the device after each attempt for analysis a posteriori. Two investigators performed each series five times.

To assess the effect of visualization techniques on experimental outcomes, a series of scapula drilling experiments were conducted using two AR guidance visualization techniques: superposed and juxtaposed. Superposed navigation placed the AR drilling guidance at the drilling site upon the glenoid surface, while juxtaposed navigation allowed the investigator to manually shift the AR guidance to a position adjacent to the scapula phantom. To eliminate errors associated with registration, all phantoms were preregistered to the IR tracker. Two investigators performed each series three times.

As no human or animal subjects or material were included as part of this work, approval was not required from the institutional review board.

### Data analysis

Pin placement outcomes were assessed through 3D scanning of the scapula with the fixed pin in place. From these scans, the pin’s entry point and direction with respect to the glenoid anatomical basis were determined. The difference between the achieved and planned ground-truth entry point and direction is reported as directional errors (inclination and version) [$^\circ$] and as positional errors (anterior-posterior and inferior-superior) [mm].

To assess the tracking performance, logged transforms for the phantom’s two IR trackers were transformed into the glenoid basis. From this, their difference is reported as displacement [mm] and rotation [$^\circ$].

To assess registration performance, the logged registration was used to transform the planned trajectory into the ground-truth glenoid coordinate system. From this, the difference between the registered and ground-truth entry point and direction is reported as displacements [mm] and rotation [$^\circ$]. The number of registration attempts is also reported.

## Results

### Registration and tracking

Of the 10 registration trials, none required refinement attempts by the investigator before they were visually satisfied with the registration quality.

**Fig. 6:**
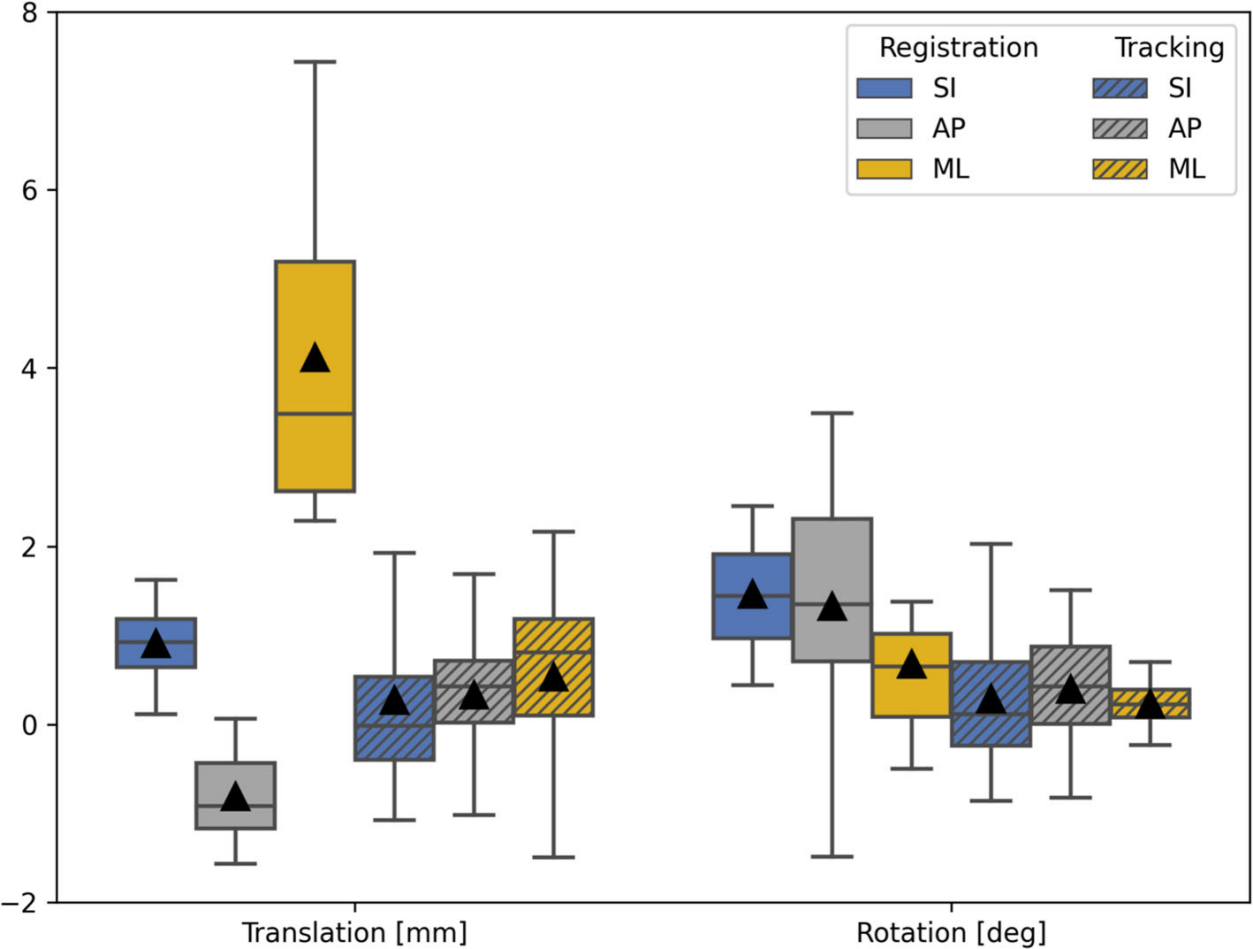
System errors for registration of planned trajectory (*n* = 10) and tracking performance (*n* = 448) along superior-inferior (SI), anteriorposterior (AP), and medial-lateral (ML) glenoid axii. Translation reported in [mm] and rotation in [deg]

Registration and tracking error are illustrated in Fig. 6, with descriptive statistics tabulated in Table 1. Mean registration error magnitudes were 4.32 mm ± 1.75 mm and ${2.56^\circ \pm 0.82^\circ }$ over the AP, SI, and ML axes, and 1.29 mm ± 0.49 mm and ${2.33^\circ \pm 0.84^\circ }$ over only the AP and SI axes. Mean tracking error magnitudes were 1.47 mm ± 0.69 mm and ${0.92^\circ \pm 0.50^\circ }$ over all three axes.

**Table 1:** Summary of system tracking and registration errors along superior-inferior (SI), anteriorposterior (AP), and medial-lateral (ML) glenoid axii.

Metric	Series	SI	AP	ML
Translation (mm)	Tracking	0.27 ± 0.95	0.33 ± 0.63	0.53 ± 0.94
	Registration	0.91 ± 0.43	$-$ 0.81 ± 0.49	4.12 ± 1.70
Rotation ($^\circ$)	Tracking	0.29 ± 0.68	0.39 ± 0.53	0.23 ± 0.22
	Registration	1.47 ± 0.65	1.33 ± 1.35	0.67 ± 0.81

### Pin placement outcomes

**Fig. 7:**
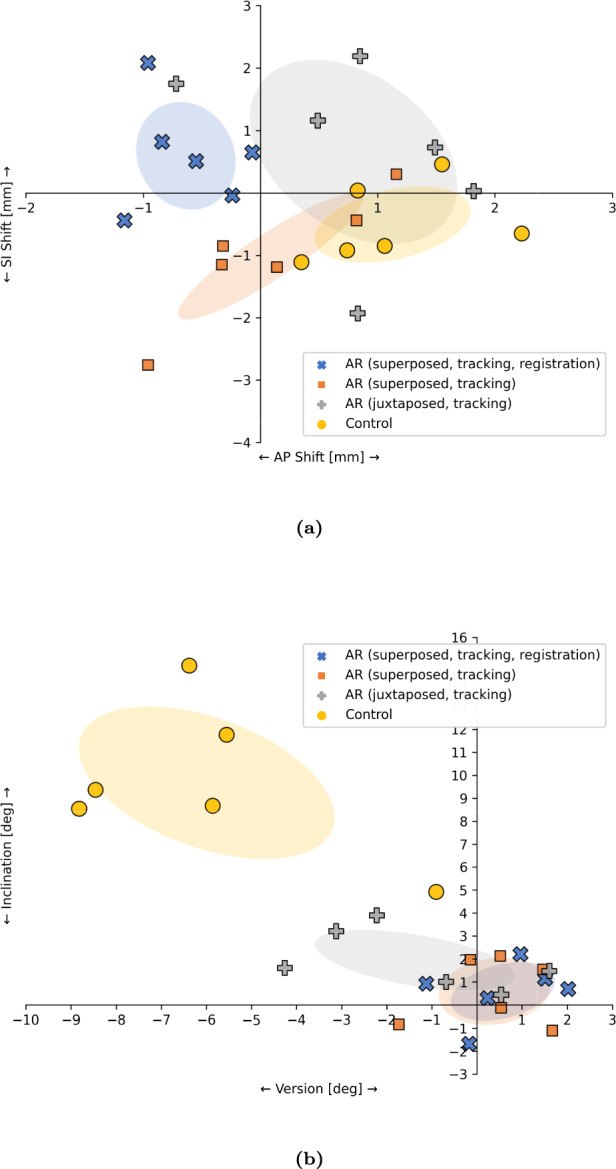
Pin placement outcomes with respect to planning. **a** Anterior–posterior (AP) and superior–inferior (SI) shift. **b** Version and inclination. Standard deviations indicated by ellipses

Pin placement outcomes are illustrated in Fig. 7, with descriptive statistics tabulated in Table 2. Error magnitudes obtained from the end-to-end AR-guided pin placements were 1.06 mm ± 0.64 mm and ${1.66^\circ \pm 0.65^\circ }$, and in the control 1.41 mm ± 0.47 mm and ${11.57^\circ \pm 3.38^\circ }$. No statistical difference between their entry point error was observed (Wilcoxon signed rank: *n* = 6, *w* = 6, *p* = 0.44); however, directional errors were significantly larger in the control (Wilcoxon signed rank: *n* = 6, *w* = 0, *p* = 0.03).

Outcome magnitudes for superposed and juxtaposed, preregistered, and AR navigated techniques were 1.39 mm ± 0.70 mm and ${1.80^\circ \pm 0.56^\circ }$, and 1.85 mm ± 0.34 mm and ${2.93^\circ \pm 1.63^\circ }$, respectively. No statistical difference in pin placement outcomes was observed with respect to entry point (Wilcoxon signed rank: *n* = 6, *w* = 4, *p* = 0.22) or direction (Wilcoxon signed rank: *n* = 6, *w* = 5, *p* = 0.31).

**Table 2:** Summery of pin placement outcomes in both the end-to-end augmented reality (AR) and control techniques as well as the superposed and juxtaposed AR-navigated experiments; superposed navigation (sup), juxtaposed navigation (jux); errors include registration (reg) and tracking (track)

Series	Inclination ($^\circ$)	Version ($^\circ$)	AP Shift (mm)	IS Shift (mm)
AR (sup, reg, track)	0.60 ± 1.18	0.58 ± 1.05	$-$ 0.64 ± 0.39	0.60 ± 0.79
AR (jux, track)	1.94 ± 1.22	$-$ 1.36 ± 2.04	0.79 ± 0.81	0.65 ± 1.35
AR (sup, track)	0.59 ± 1.33	0.39 ± 1.13	0.08 ± 0.72	$-$ 1.01 ± 0.93
Control	9.67 ± 3.04	$-$ 6.00 ± 2.59	1.13 ± 0.61	$-$ 0.50 ± 0.56

## Discussion

Though tracking errors were small, registration errors were found to be comparatively large. In particular, rotation and translation magnitudes were above acceptable thresholds of 2 mm and ${2}^\circ$ [35]. It is suspected that the small glenoid surface, over which sparse points were collected, provided limited geometric features, and together with a variable stylus handling technique and potential tool-tip shift along its long axis contributing to this outcome. Given the described AR guidance, only AP and SI registration errors were relevant, as rotation about or shift along the ML axis would not have affected drilling outcomes, fortunately, as positional registration quality along that axis was clearly biased.

Surprisingly, the observed end-to-end errors of the AR workflow—comprising contributions of registration, tracking, and user performance when provided with AR guidance—were lower than the registration error alone. This seems to indicate some compensation by the user, based on their experience and anatomical knowledge, during AR-guided drilling. The thresholds used for the go/no-go color feedback allowed for as much as 2 mm and ${2}^\circ$ of error or compensation of registration. Testing during development indicated that this margin was appropriate, as lower thresholds were difficult to use while higher thresholds induced inaccuracy. The position of the AR guidance during drilling, superposed or juxtaposed, did not statistically affect drilling outcomes. The literature has indicated that alternative user interfaces could be more appropriate for navigation [36] and should be investigated in the TSA use case.

Across all outcome metrics for glenoid pin placement, end-to-end AR guidance yielded results in line with those reported for patient-specific 3D printed guides (1.2 mm and ${2.9}^\circ$) [7]. This supports the efficacy of off-the-shelf AR hardware coupled with dedicated navigation software as a viable tool for computer-guided pin placement. Moreover, the AR-navigated use case relies on the same preoperative planning performed during the design of patient-specific 3D printed guides, though execution of the plan can be performed in AR, reducing or eliminating lead times related to their logistics.

In addition to this parity, the proposed technique outperforms published literature in CAN for TSA using similar AR hardware [23, 28, 29, 37]. We attribute this to our tracking and drift correction approach. Indeed, previous studies have shown that infrared tracking increases the stability of tool tracking and improves drift correction of registration in AR applications to below 2 mm and ${2}^\circ$ [12, 31, 32]. The obtained tracking performance reflects this literature and provides one explanation for the superior results. Additionally, through the described tracking pipeline, using data solely from the IR reflectivity map, the results were comparable to those obtained through computationally expensive photogrammetric techniques [30], while achieving on-device real-time performance.

The end-to-end AR workflow improved pin placement orientation compared to the control free-handed technique; however, it did not improve entry-point error. These findings, as well as the relatively large angular errors obtained from the control group, are reflected in literature comparing navigated vs un-navigated glenoid positioning [8]. This highlights the difficulty in judging an optimal drilling angle into the scapula but the ease in finding the glenoid’s center given only visual feedback through the surgical window. It may be speculated that had the planned entry point not been at the glenoid’s center, the control group may not have achieved equivalence along any outcome metric, while AR would have performed similarly. Alternatively, it may just be that determining the entry point visually is simple, irrespective of whether it’s at the center of the glenoid or not, and is a point for later research.

This study’s use of Microsoft’s HoloLens 2 AR-HMD raises several important limitations for the technology’s role as a surgical navigation tool. Although the device has become a standard in medical AR research since its launch and has commercially been granted 510K FDA approval in several commercial navigation systems—though often in limited roles such as viewing preoperative planning alongside a patient—the display technology suffers from two important issues. First, a disparity between focal planes of the operative field and AR navigation on the HMD display often leads to difficulty in focusing on both spaces. This was found to be most noticeable during registration tasks, as more complex data was shown superposed onto reality. Secondly, while the AR-HMD does construct a surface mesh of the environment in order to occlude AR visualization naturally, an important depth estimation cue, its coarseness and latency often lead to unnatural visual artifacts in the THA use case, being obvious in Fig. 2b. It was found that a visual “less is more” approach to navigation helped attenuate these effects. This minimization of visual feedback has consequences at every stage of the surgical workflow, e.g., registration quality assurance and drill alignment. Therefore, their design is certainly worth formally and qualitatively assessing given limitations of the chosen hardware.

While the use of phantom scapulae lacking arthritic deterioration and cartilage tissues, as encountered in clinical and cadaver studies, the results are seen as translatable to real-world conditions. The use of a fine-tipped infrared-tracked stylus could sufficiently penetrate such tissues towards underlying bone during registration, as is done in conventional commercial systems. Moreover, the drilling plan, being patient-specific and defined preoperatively along with the segmented scapula model used for plan registration, is similarly navigated regardless of arthritic bone state. Despite this, follow-up testing using the proposed navigation techniques should incorporate cadaver testing in order to better simulate the drilling feedback through bone, as the foam core Sawbones models did not accurately capture this.

The primary limitations of this study are the small sample size of both the number of scapula (*n* = 12) and the number of participants (*n* = 2). This limits the statistical power of conclusions drawn. Lastly, the end-to-end AR guidance was not directly compared with state-of-the-art commercial computer navigation techniques, nor emerging technologies such as robotic-assisted surgery which are demonstrating comparable performance [38].

## Conclusion

A phantom study evaluated the effectiveness of an adapted off-the-shelf augmented reality device to navigate glenoid pin placement in shoulder arthroplasty. It was shown that the direction of the pin placement, with respect to the preoperative planning, was improved when compared to the current free-handed technique. The results achieved state-of-the-art performance for planned pin placement and were comparable to those based on patient-specific 3D printed guides.
